# Abdominal Wall Reconstruction After Extirpation of a 140-Pound Primary Ovarian Mucinous Adenocarcinoma

**DOI:** 10.7759/cureus.35542

**Published:** 2023-02-27

**Authors:** Mamtha S Raj, Karthik Pittala, Sean J Wallace, Randolph Wojcik Jr.

**Affiliations:** 1 Department of Surgery, Division of Plastic & Reconstructive Surgery, Yale New Haven Health, New Haven, USA; 2 Division of Plastic & Reconstructive Surgery, Lehigh Valley Health Network, Allentown, USA; 3 Medicine, University of South Florida Morsani College of Medicine, Tampa, USA

**Keywords:** women’s health, ovarian mass, ovarian mucinous adenocarcinoma, abdominal wall body contouring, abdominal wall reconstruction

## Abstract

Ovarian cancer is a feared diagnosis for women and clinicians alike. Ovarian mucinous adenocarcinoma is a unique subset of ovarian cancer. As a primary tumor, massive ovarian masses, and more specifically mucinous adenocarcinomas, have been infrequently reported in the medical literature. Team approaches to massive tumor extirpations are essential, as patients often require the expertise of various subspecialists including, but not limited to, gynecologic-oncologists, general surgeons, and plastic and reconstructive surgeons. Here we present a case of a 71-year-old woman with a massive, incapacitating pelvic mass, later found to be a primary ovarian mucinous adenocarcinoma. Once medically optimized, a multi-service team approach was utilized for tumor extirpation and abdominal wall reconstruction. Involved surgical services included Gynecologic-Oncology, General Surgery, and Plastic and Reconstructive Surgery. Exploratory laparotomy for tumor extirpation, hysterectomy, bilateral salpingo-oophorectomy, omentectomy, peritoneal stripping, bilateral inguinal lymphadenectomy, and appendectomy was performed. Extensively thin, devascularized, and attenuated abdominal wall fascia that was adherent to the tumor was removed. The abdominal wall defect was reconstructed and reinforced with inlay and overlays of biologic monofilament mesh. Inverted-T of the vertical and horizontal skin components was performed in a tailor-tacking fashion, assuring the maintenance and protection of the abdominal skin flap vascularity through utilizing the Huger Zones of perfusion. Pathology revealed a stage IA grade 2 mucinous adenocarcinoma of the ovary without evidence of metastasis. No adjuvant therapies were required. The tumor’s weight was 140 pounds, and its dimensions were 63 x 41 x 40 cm. It is our hope that presenting this experience will raise awareness of this spectrum of diseases and allow for earlier diagnoses and treatments, as well as exemplify the virtues of a team-based approach in the successful extirpation and subsequent reconstruction of the abdominal wall and skin.

## Introduction

Ovarian cancer is the seventh most common cancer and the eighth most common cause of cancer death in women [1,2]. Ovarian cancer has several histologic subtypes, with high-grade serous ovarian cancer being the most common, accounting for 65% of all cases. Mucinous ovarian adenocarcinoma (MOC) is a unique histologic subtype of ovarian cancer, which accounts for 3% of all epithelial ovarian cancers. Tian et al. reported a 100% five-year survival in a small cohort of patients with primary MOC [3]. Conversely, stage III and IV MOCs carry poorer prognoses. Patients with metastatic mucinous disease have a median survival rate of 12 months in advanced stages [4-6].

This article was previously presented as a meeting abstract at the 2018 Keystone Chapter of the American College of Surgeons Meeting in Hershey, PA, on November 2, 2018, and at the 2018 Robert H. Ivy Pennsylvania Plastic Surgery Society Meeting in Hershey, PA, on April 21, 2018.

## Case presentation

**Figure 1:**
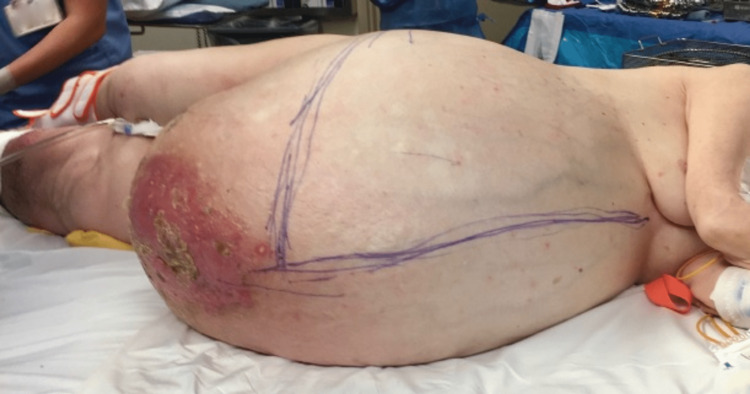
Pre-operative image of the rotund abdominal wall

**Figure 2:**
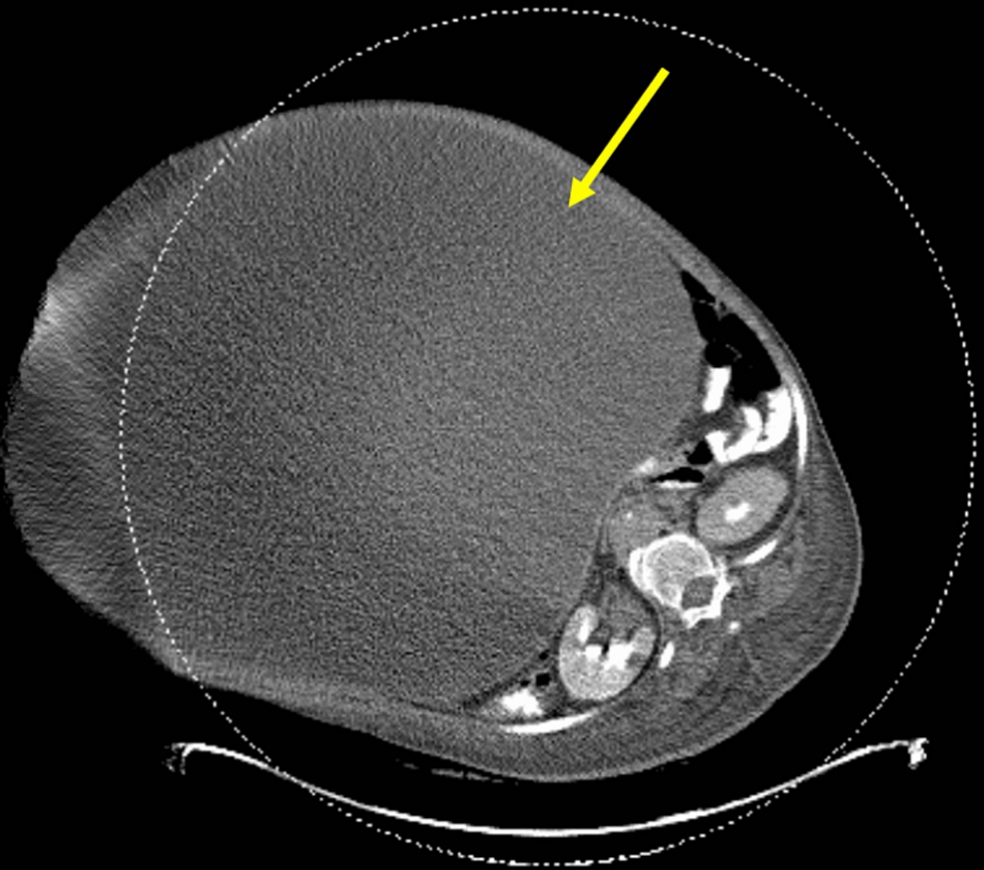
Computed tomography showing the large intra-abdominal mass

The patient was a 71-year-old woman found to have a massive and incapacitating pelvic mass of unknown etiology for at least a 15-year timeframe. She initially presented to the hospital with left leg pain, cellulitis, and worsening shortness of breath, but was noted to have significant abdominal distension and increased weight gain over this time period. Complicating associated comorbidities included deep venous thrombosis (DVT), bilateral hydronephrosis, and morbid obesity with a body mass index (BMI) of 65.3 (Figure 1). Physical examination revealed a morbidly obese female with labored breathing requiring supplemental oxygen, a rotund, distended, tender, and cellulitic-appearing abdominal wall with lichenification of the overlying skin, and significant bilateral lower extremity edema. Computed tomography revealed a >60 cm abdominopelvic mass with associated bilateral hydronephrosis (Figure 2). Ultrasound of the lower extremities showed left femoral and popliteal DVT. Pre-operative risk stratification and medical optimization were performed. She was deemed to be a low-risk surgical candidate with a Revised Cardiac Risk Index of 0.9%. Systemic anticoagulation was initiated, and a Greenfield vena cava filter placement was performed prior to surgery as part of her optimization.

Once medically optimized, an interdisciplinary team discussion and approach were utilized for the planning of oncologic ablation and abdominal wall reconstruction. Involved surgical services included Gynecologic-Oncology, General Surgery, and Plastic and Reconstructive Surgery.

**Figure 3:**
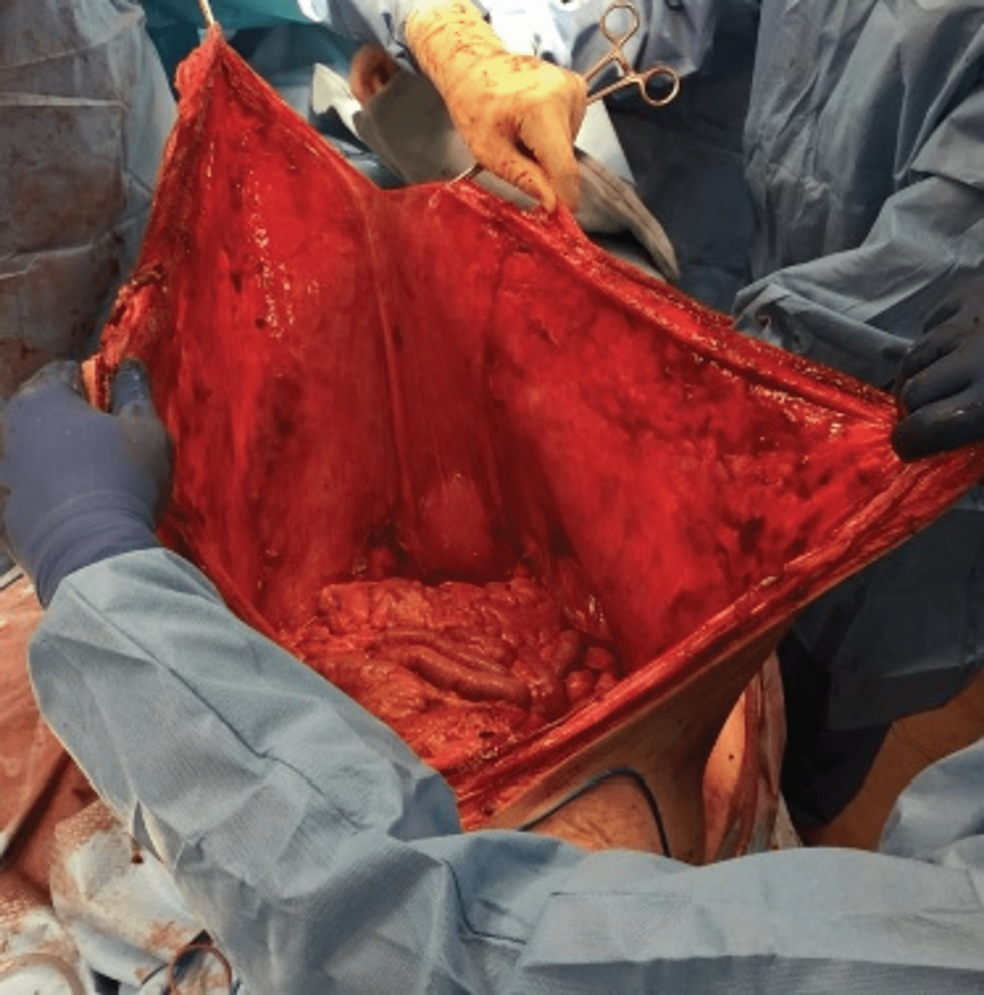
Attenuated abdominal wall fascia

**Figure 4:**
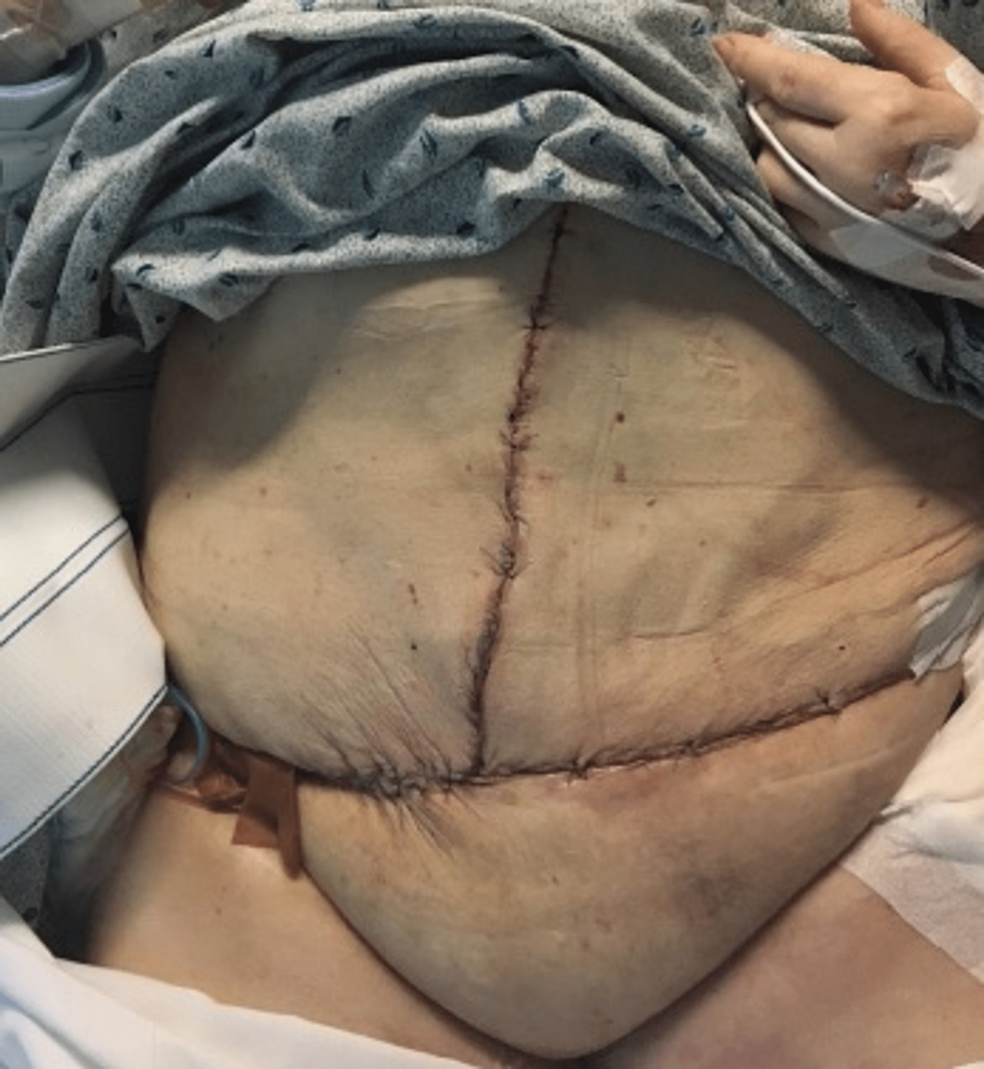
Closed and contoured abdominal wall and skin

With the patient unable to lay supine secondary to the size of the mass, she was positioned in the left lateral decubitus position with two operating room tables placed side-by-side. Skin resection and exposure of the abdominal wall were marked and performed in both horizontal (90 x 20 cm) and vertical (60 x 20 cm) directions, assuring to maintain and protect perforating blood vessels supplying zones 1, 2, and 3 of the abdominal wall, as first described by Huger et al. in 1979 [7]. With great caution, entrance was gained into the peritoneal cavity, which revealed a gigantic mass with significant adherence to the abdominal wall fascia and musculature. Complete extirpation required en bloc resection of the mass with a portion of the fascia and abdominal musculature. Once the mass was circumferentially free from the peritoneal cavity, it was rolled from the operating field onto a sterilely draped cart and sent for permanent pathology. The patient was then transitioned to the supine position for the remaining portion of the procedure. Hysterectomy, bilateral salpingo-oophorectomy, omentectomy, bilateral inguinal lymphadenectomy, and appendectomy were performed. Due to the extremely thin and de-vascularized nature of the extensively attenuated abdominal wall fascia, all non-viable portions were removed. The posterior rectus sheath was then reapproximated with polydioxanone suture (PDS). As reinforcement, an inlay of biologic resorbable monofilament mesh was placed and secured circumferentially to the posterior rectus sheath. The anterior rectus sheath was then reapproximated with PDS suture and reinforced with an overlay of biologic resorbable monofilament mesh that was secured circumferentially to the anterior rectus sheath (Figure 3). Perforating blood vessels adhering to Huger zones of the remaining abdominal wall skin were then identified, dissected, and protected to assure perfusion of the large skin flaps and complex closures of the vertical and horizontal skin components in a tailor-tacking fashion over multiple drains (Figure 4). 

Secondary to massive volume shifts intraoperatively, a large volume of crystalloids, colloids, blood products, and vasopressors were required throughout her procedure. Post-operatively, she remained intubated and monitored in the intensive care unit due to hemodynamic instability and ventilator dependence. She was volume-resuscitated and subsequently extubated, and her diet and activity slowly advanced. An abdominal binder was worn at all times for 12 weeks post-operatively, only being removed for hygienic purposes. On postoperative day 20, she was discharged to a skilled nursing facility for continued rehabilitation with lifting restrictions of nothing greater than 10 pounds until eight weeks post-operatively.

**Figure 5:**
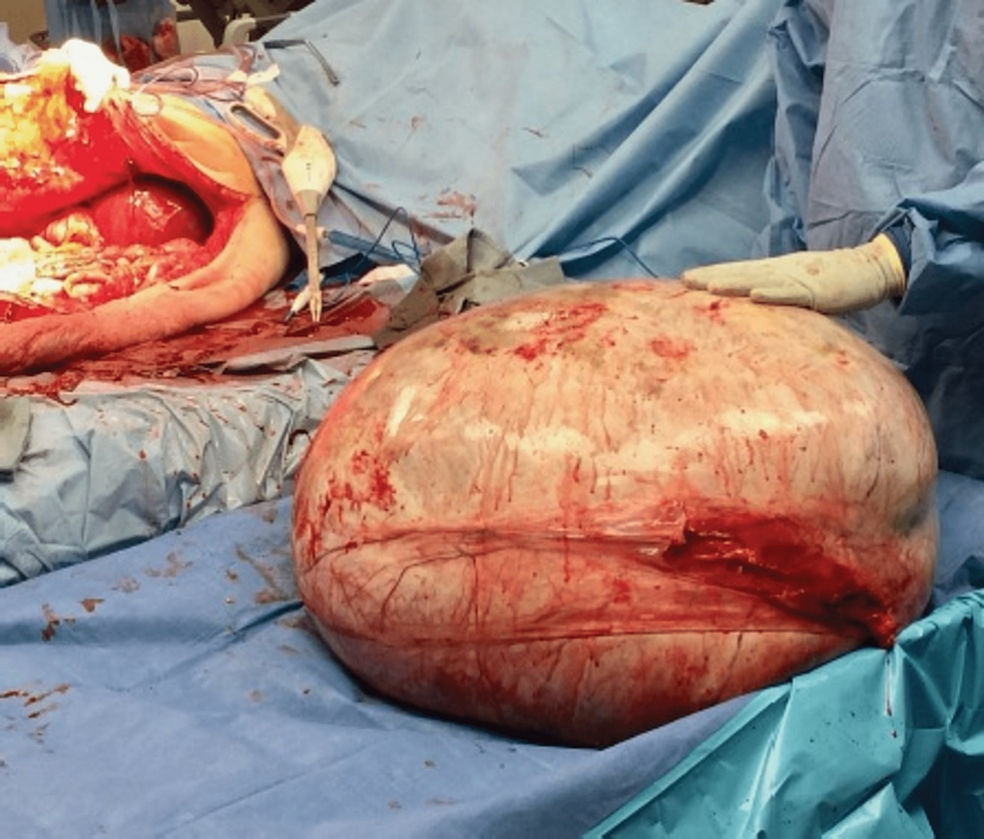
Ovarian mass after extirpation

Pathology revealed a stage IA grade 2 mucinous adenocarcinoma of the ovary without evidence of metastatic carcinoma. The final tumor weight and dimensions registered 140 pounds and 63 x 41 x 40 cm, respectively (Figure 5). The patient’s left adnexal specimen was positive for immunohistochemical markers CK7 and CK20. She was followed up closely as an outpatient by all involved surgical services. A cancer antigen 125 (CA-125) level of 4.8 U/mL (normal: 0.0-38.1 U/mL) and carcinoembryonic antigen (CEA) level of 0.9 ng/mL (normal: 0.0-3.0 ng/mL) provided evidence of her cancer remission. No dehiscence or wound-healing problems arose in the postoperative period. She returned to her daily activities and no adjuvant oncologic therapies were required.

## Discussion

Mucinous adenocarcinomas typically arise from the gastrointestinal tract, endometrium, and/or endocervix [3]. Although ovarian cancer is the most lethal gynecologic malignancy, the mucinous adenocarcinoma subtype is a small part of the histologically benign variant of ovarian neoplasms [8]. Overall, the incidence of primary ovarian mucinous adenocarcinoma among patients diagnosed with ovarian cancer showcases its rare presentation, with studies mentioning ranges from 1.6% to 4.9%. These tumors typically present in young female adults, with a mean size of 18 cm but are known to grow large and occlude major parts of the abdominopelvic space, as seen in our case [9].

The distinction between primary and metastatic mucinous ovarian cancers is made on histologic examination and immunohistochemical (IHC) staining. Common IHC markers include cytokeratin 7 (CK7), CK20, CEA, carbohydrate antigen 19.9 (CA19.9), caudal-type homeobox 2 (CDX2), and CA-125 [10]. Of these, CEA and CA-125 are commonly used in monitoring for the recurrence of disease [4]. Team approaches to massive tumor extirpations are essential as patients often require the expertise of various subspecialists including, but not limited to, gynecologic-oncologists, general surgeons, and plastic and reconstructive surgeons. In this report, we present the case of a patient who underwent extirpation of a 140-pound primary ovarian mucinous adenocarcinoma and subsequently underwent abdominal wall reconstruction.

Extirpation and abdominal wall reconstruction were performed with great improvement in the patient’s health, ambulatory status, and reclamation of her independence. Pathology revealed stage IA grade 2 mucinous adenocarcinoma of the ovary that was negative for metastatic disease. This report presents a case of larger primary ovarian mucinous adenocarcinomas present in the current medical literature. The patient’s positivity for CK7 and CK20 confirms the diagnosis of primary ovarian mucinous adenocarcinoma, while monitoring of her levels of CEA and CA-125 normalcy signifies her oncologic remission. This patient is now in clinical remission and has resumed her normal daily function.

## Conclusions

Mucinous ovarian adenocarcinoma is a rare subtype of ovarian cancer that can present with an expanding, massive tumor, as seen with the current case. The tumor was positive for immunohistochemical markers CK7 and CK20, supporting its primary origin. Recurrence was monitored with CEA and CA-125 levels, which remained within normal limits post-operatively throughout multiple follow-up appointments. Although the surgery proceeded without complications and the patient recovered uneventfully, this case is an important contribution to the literature through further characterization of this tumor type, confirmation of histology and immunohistochemistry, and break-down of surgical techniques. Interdisciplinary team-based discussion and approaches are essential in patients presenting with complex surgical problems, as patients often require the expertise of various medical and surgical subspecialists. Only by working together can we provide the best outcomes for such patients.
